# Diagnostic value of symptoms for pediatric SARS-CoV-2 infection in a primary care setting

**DOI:** 10.1371/journal.pone.0249980

**Published:** 2021-12-13

**Authors:** Chien-Hsiang Weng, Wesley Wing Wah Butt, Meredith B. Brooks, Claudia Clarke, Helen E. Jenkins, Sabina D. Holland, Silvia S. Chiang

**Affiliations:** 1 Department of Family Medicine, Brown University Warren Alpert Medical School, Providence, RI, United States of America; 2 Providence Community Health Centers, Providence, RI, United States of America; 3 Department of Global Health and Social Medicine, Harvard Medical School, Boston, MA, United States of America; 4 Department of Biostatistics, Boston University School of Public Health, Boston, MA, United States of America; 5 Department of Pediatrics, Brown University Warren Alpert Medical School, Providence, RI, United States of America; 6 Center for International Health Research, Rhode Island Hospital, Providence, RI, United States of America; The University of Hong Kong, CHINA

## Abstract

**Purpose:**

To evaluate the diagnostic value of symptoms used by daycares and schools to screen children and adolescents for SARS-CoV-2 infection, we analyzed data from a primary care setting.

**Methods:**

This cohort study included all patients ≤17 years old who were evaluated at Providence Community Health Centers (PCHC; Providence, U.S.), for COVID-19 symptoms and/or exposure, and received SARS-CoV-2 polymerase chain reaction (PCR) testing between March-June 2020. Participants were identified from PCHC electronic medical records. For three age groups– 0–4, 5–11, and 12–17 years–we estimated the sensitivity, specificity, and area under the receiver operating curve (AUC) of individual symptoms and three symptom combinations: a case definition published by the Rhode Island Department of Health (RIDOH), and two novel combinations generated by different statistical approaches to maximize sensitivity, specificity, and AUC. We evaluated symptom combinations both with and without consideration of COVID-19 exposure. Myalgia, headache, sore throat, abdominal pain, nausea, anosmia, and ageusia were not assessed in 0–4 year-olds due to the lower reliability of these symptoms in this group.

**Results:**

Of 555 participants, 217 (39.1%) were SARS-CoV-2-infected. Fever was more common among 0–4 years-olds (p = 0.002); older children more frequently reported fatigue (p = 0.02). In children ≥5 years old, anosmia or ageusia had 94–98% specificity. In all ages, exposure history most accurately predicted infection. With respect to individual symptoms, cough most accurately predicted infection in <5 year-olds (AUC 0.69) and 12–17 year-olds (AUC 0.62), while headache was most accurate in 5–11 year-olds (AUC 0.62). In combination with exposure history, the novel symptom combinations generated statistically to maximize test characteristics had sensitivity >95% but specificity <30%. No symptom or symptom combination had AUC ≥0.70.

**Conclusions:**

Anosmia or ageusia in children ≥5 years old should raise providers’ index of suspicion for COVID-19. However, our overall findings underscore the limited diagnostic value of symptoms.

## Introduction

SARS-CoV-2 has caused COVID-19 in 35 million people in the United States (U.S.) and close to 15% of the population in the state of Rhode Island as of early August 2021 [1]. Children and adolescents account for 14.2% of reported COVID-19 cases in the U.S [2]. Most children and adolescents have mild symptoms and are managed as outpatients [3–5]; however, few clinical studies of pediatric COVID-19 have been conducted in primary care settings [6–8]. A clearer understanding of the diagnostic value of symptoms may have implications for the symptom screening approaches used in daycares and schools for identifying children and adolescents with possible SARS-CoV-2 infection.

On April 1, 2020, the Rhode Island Department of Health (RIDOH) began recommending SARS-CoV-2 testing for anyone with exposure to or symptoms of COVID-19 [9]. Concurrently, local hospitals implemented pre-admission and pre-procedure COVID-19 screening. When daycares and schools in Rhode Island reopened after having closed for several months, RIDOH recommended the use of a probable case definition to screen daycare and school attendees for COVID-19 symptoms. According to the RIDOH criteria, a probable COVID-19 case has one of the following: new cough, shortness of breath, or anosmia or ageusia. A case also qualifies as probable COVID-19 by having at least two of the following: fever, chills, myalgia, headache, sore throat, nausea or vomiting, diarrhea, fatigue, or new congestion or rhinorrhea [10].

To estimate the accuracy of symptoms for identifying pediatric SARS-CoV-2 infection, we conducted this cohort study of all patients ≤17 years old who were evaluated at Providence Community Health Centers (PCHC), for COVID-19 symptoms and/or exposure, and received SARS-CoV-2 polymerase chain reaction (PCR) testing between March-June 2020 for symptoms, exposure, and/or pre-procedure screening. We assessed the test characteristics of individual symptoms and three symptom combinations: the RIDOH probable case definition [10], which is used to screen students and daycare attendees for COVID-19, and two novel combinations generated by statistical approaches to maximize sensitivity and area under the receiver operating curve (AUC). As a secondary aim, we evaluated epidemiological predictors of SARS-CoV-2 infection in the cohort.

## Methods

### Setting

This retrospective cohort study took place between March 20-June 22, 2020 at PCHC, a network of ten clinics that provide primary care, urgent care, and specialty services. PCHC serves approximately 60,000 patients of all ages, who are predominantly Hispanic. Ninety percent of patients have household incomes under 200% of the federal poverty level [11], Before the study start date, PCHC clinicians implemented a standardized template to document symptoms and exposure history of patients under evaluation for COVID-19. At the time of the study, the only known circulating strain of SARS-CoV-2 was the wildtype.

### Participants

We identified all PCHC patients who received SARS-CoV-2 reverse transcriptase polymerase chain reaction (RT-PCR) testing on a nasopharyngeal sample on or before June 22, 2020, and were 17 years old or at the time of the test. We included patients whose exposure and symptoms were evaluated either before or after PCR testing. The latter group consisted of patients tested in an emergency department or who underwent pre-procedure or pre-admission COVID-19 screening, as long as their PCR result was documented in their PCHC chart and they were evaluated with the standardized template during a follow-up visit. During the clinical evaluation, PCHC providers used a standardized template to verbally query symptoms from patients and/or their caregivers. Myalgia, headache, sore throat, abdominal pain, nausea, and anosmia or ageusia were not assessed in children 0–4 years old due to their lower ability to report these symptoms.

### Data collection and variables

Three authors (CW, WB, CC) manually abstracted the following variables: age, sex, self-reported race/ethnicity, insurance status, body mass index (BMI)-for-age percentile, history of asthma or allergic rhinitis, COVID-19 exposure history, symptoms present through the clinical evaluation (or test date if PCR testing preceded the evaluation), type of encounter that led to SARS-CoV-2 testing (PCHC primary care, PCHC urgent care, emergency department, procedure, or hospital admission), and time between symptom onset and PCR test. Known COVID-19 exposure was self-reported and defined as contact with a confirmed or suspected COVID-19 patient ≤14 days prior to SARS-CoV-2 testing. On the standardized template, the following symptoms were marked as present or absent: new cough, dyspnea, new congestion/rhinorrhea, myalgia, fever ≥100.4°F, headache, sore throat, abdominal pain, nausea, vomiting, diarrhea, anosmia or ageusia, and fatigue.

We categorized participants into age groups corresponding roughly with U.S. educational stages: 0–4 years (daycare/preschool), 5–11 years (elementary school), and 12–17 years (middle/high school). Race/ethnicity was grouped into Hispanic, non-Hispanic (NH) Black, NH White, and NH other (Asians, other Pacific Islanders, more than one race, and unknown). We used CDC definitions to categorize BMI-for-age percentile [12].

### Statistical analysis

We compared demographic and clinical characteristics between SARS-CoV-2-infected and uninfected participants using the chi-squared test. Variables that differed between the two groups at a significance level of p<0.2 were entered into a multivariable model. We checked for interactions on the multiplicative scale between known COVID-19 exposure and all other covariates.

For each age group, we calculated the sensitivity, specificity, and AUC of COVID-19 exposure history and each symptom for identifying SARS-CoV-2 infection. Myalgia, headache, sore throat, abdominal pain, nausea, and anosmia or ageusia were not assessed in children 0–4 years old due to their lower ability to report these symptoms. We then evaluated the diagnostic value of three symptom combinations: (1) the RIDOH probable case definition [10]; (2) a combination generated by a backward elimination approach; and (3) a combination generated by classification and regression tree (CART) analysis. As we did not collect information on chills, we excluded this symptom in our analysis of the RIDOH criteria. We evaluated the test characteristics of the three symptom combinations both with and without consideration of COVID-19 exposure.

For each age group, we used a backward elimination approach to generate a symptom combination that maximized specificity without sacrificing sensitivity. First, we calculated the sensitivity, specificity, and AUC if any of the symptoms were present (the baseline combination). Then, we manually removed symptoms one at a time, in order of ascending AUC. Symptoms with the same AUC were removed in order of ascending sensitivity. We selected the combination with the highest specificity but the same sensitivity as the baseline combination.

We used CART analysis to identify the symptoms that best predicted SARS-CoV-2 infection in each age group. In CART analysis, measures of predictive importance were assigned to each symptom, entailing both marginal and interaction effects involving this variable. The data set was then split into increasingly homogenous sub-groups, using improvement in the Gini gain score, to identify the explanatory variable that gave the best discrimination between the two outcome classes (COVID-19 vs. no COVID-19). Maximal trees were created and then pruned based on relative misclassification costs, complexity, and parsimony. Ten-fold cross-validation was performed, in which the whole data set was randomly split into learning and test data sets. CART analysis was then applied to determine model performance and predictive accuracy in these test sets, removing the need for a validation data set. We calculated discriminatory properties of having at least one of the most important symptoms identified as nodes on the final derived trees for each age group.

To determine the impact of recall bias from applying the standardized template after PCR testing, we performed a sensitivity analysis restricted to participants who were evaluated for exposure and symptoms before testing. We decided *a priori* to assess the diagnostic value of symptom combinations only if there were meaningful differences in AUCs of exposure history and individual symptoms.

With respect to handling missing data in the analyses, twenty-two (4.0%) participants with unknown race/ethnicity were grouped into the “other” group and included in all analyses. BMI-for-age percentile—which is measured in children at least two years old—was missing for 128 (23.1%) participants, 96 of whom were younger than two years. These participants were excluded from comparisons of BMI between SARS-CoV-2-infected and uninfected children only. Four (0.7%) participants had no data for one symptom; they were excluded from regression models that examined the association between the number of presenting symptoms and SARS-CoV-2 infection, as well as from calculations of sensitivity, specificity, and AUC for the missing symptom only.

Analyses were conducted using R version 3.5.1 (R Statistical Computing, Vienna, Austria) and Salford Systems Data Mining and Predictive Analytics Software version 8.0 (Salford Systems, San Diego, California, U.S.). Sensitivity, specificity, and AUC estimates were calculated with the reportROC package for R [13].

### Ethics

The PCHC Human Subjects Review Committee approved this study and waived informed consent.

## Results

Before June 22, 2020, SARS-CoV-2 PCR was performed in 803 individuals <18 years of age who were registered as PCHC patients. We included 555 (69.1%) who were evaluated using the standardized template. PCHC clinicians assessed 506 (91.2%) participants in primary care and five (0.9%) in urgent care prior to PCR testing. Forty-four (7.9%) participants were assessed by PCHC clinicians after pre-procedure screening (n = 10), hospital admission (n = 21), and emergency room visit (n = 13). The 248 excluded patients were tested at a PCHC specialty clinic or a non-PCHC facility without subsequent evaluation using the standardized template (S1 Table).

Median age was 9 (IQR: 3.5–15) years; 293 (52.8%) were female, 459 (82.7%) were Hispanic, and 37 (6.7%) were uninsured (Table 1). Of the 555 participants, 283 (51.0%) had known COVID-19 exposure and at least one symptom; 183 (33.0%) had at least one symptom but no known exposure; 56 (10.1%) had known exposure but no symptoms; and 33 (5.9%) participants had neither symptoms nor known exposure.

**Table 1:** Epidemiological characteristics of study population.

	n (%)			
	All (n = 555)	Uninfected (n = 338)	Infected (n = 217)	p-value
Age	9 (3.5–15)	8 (3–14)	11 (6–15)	<0.001
Female	293 (52.8)	183 (54.1)	110 (50.7)	0.43
Race/ethnicity^a^				<0.001
NH Black	26 (4.7)	21 (6.2)	5 (2.3)	
Hispanic	459 (82.7)	257 (76.0)	202 (93.1)	
NH White	30 (5.4)	24 (7.1)	6 (2.8)	
Other	40 (7.2)	36 (10.7)	4 (1.8)	
Uninsured	37 (6.7)	17 (5.0)	20 (9.2)	
BMI category^b^				0.20
Normal	206 (48.2)	127 (50.2)	79 (45.4)	
Underweight	11 (2.6)	9 (3.6)	2 (1.1)	
Overweight	77 (18.0)	40 (15.8)	37 (21.3)	
Obese	133 (31.1)	77 (30.4)	56 (32.2)	
Asthma or allergic rhinitis	110 (19.8)	70 (20.7)	40 (18.4)	0.51
Known COVID-19 exposure	339 (61.1)	150 (44.4)	189 (87.1)	<0.001
Evaluated for SARS-CoV-2 after stay-at-home order lifted	289 (52.1)	9 (3.6)	2 (1.1)	0.49

^a^Of 459 Hispanics, 2 were Asian; 13, Black; 247, White, 85, >1 race; 3, Pacific Islander; 109, Unknown. Of 40 “Other,” 9 were Asian; 8, >1 race; 1, Pacific Islander; 22, Unknown.

^b^BMI category applies only to children age ≥2 years. In addition, 18 uninfected and 14 infected children ≥2 years were missing BMI category; therefore, the percentages for the uninfected group and the infected group are calculated from 254 and 174, respectively.

Abbreviations: BMI, body mass index; aOR, adjusted odds ratio; CI, confidence interval; IQR, interquartile range; OR, odds ratio; Ref, reference category.

Ninety-seven (20.8%) of 466 symptomatic patients had unknown symptom duration prior to PCR testing. Of the remaining 369, 323 (87.5%) were tested ≤7 days of symptom onset, of whom 178 (55.1%) were uninfected, and 145 (44.9%) were infected. Of 46 (12.5%) tested >7 days of symptom onset, 22 (47.8%) were uninfected, and 24 (52.2%) were infected. Test positivity did not differ between the two groups (p = 0.44).

Two-hundred eighty-nine (52.1%) participants were tested after May 9, 2020, when stay-at-home orders (i.e., lockdown) were lifted and selected non-essential business and services–specifically, retail stores, restaurants, and places of worship–were permitted to operate with restrictions. However, working from home was still required whenever possible, and gatherings had to be limited to five people (except for funerals, which had a limit of ten people) [14].

**Fig 1:**
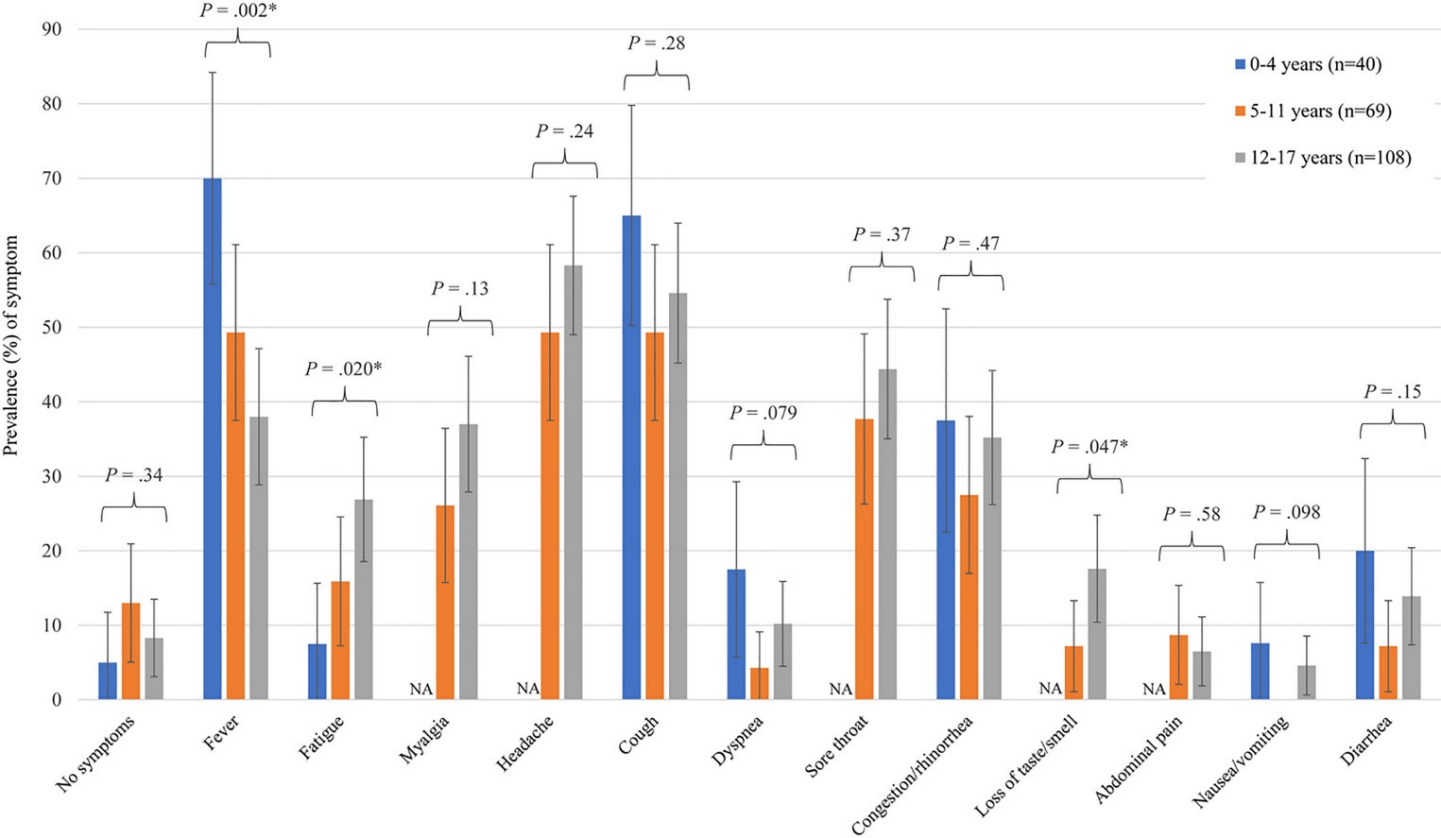
Age-stratified clinical presentation among 217 SARS-CoV-2-positive participants. Myalgia, headache, sore throat, abdominal pain, nausea, and loss of taste/smell were not assessed in children 0–4 years-old due to the lower reliability of these symptoms in this group. Bars show 95% confidence intervals. No children between the ages of 5–11 years presented with nausea/vomiting. Abbreviation: NA, not applicable.

Two-hundred seventeen (39.1%) participants had SARS-CoV-2 infection. One infected participant had neither known exposure nor symptoms. Asymptomatic infections occurred in 2/40 (5.0%) 0–4 year-olds, 9/69 (13.0%) 5–11 year-olds, and 9/108 (8.3%) 12–17 year-olds (Fig 1). Twenty-eight (12.9%) SARS-CoV-2-infected participants had only one symptom (S2 Table).

Children with a positive PCR were more likely to be older (11 vs. 8 years), have known COVID-19 exposure (87.1 vs 44.4%), be Hispanic (93.1 vs. 76.0%), and present with more symptoms (3 vs. 2 symptoms). Test positivity did not differ between participants evaluated before and after reopening. The multivariable regression analyses showed consistent results (Table 2).

**Table 2:** Epidemiological predictors of SARS-CoV-2 infection from multivariable analyses.

	Multivariable regression	
	aOR (95% CI)	p-value
Age	1.00 (0.97, 1.04)	0.88
Race/ethnicity^a^		
NH Black	Ref	
Hispanic	3.73 (1.36, 12.10)	0.016
NH White	1.91 (0.43, 8.78)	0.40
Other	0.60 (0.14, 2.75)	0.51
Uninsured	1.68 (0.76, 3.81)	0.20
Known COVID-19 exposure	8.75 (5.47, 14.46)	<0.001
Number of COVID-19 symptoms^a^	1.32 (1.18, 1.47)	<0.001

^a^COVID-19 symptoms: new cough, shortness of breath, anosmia or ageusia, fever, chills, myalgia, headache, sore throat, nausea or vomiting, diarrhea, fatigue, or new congestion or rhinorrhea.

Stratifying the 217 children with COVID-19 by age, we observed fever more frequently among children aged 0–4 years (p = 0.002). The prevalence of fatigue increased with age (p = 0.02). Adolescents 12–17 years old (p = 0.047) were more likely to present with anosmia or ageusia compared to children aged 5–11 years (Fig 1; S3–S9 Tables).

In all age groups, known COVID-19 exposure alone had the highest AUC for identifying SARS-CoV-2 infection. No individual symptom or symptom combination had AUC >0.7 (Tables 3–5). When exposure history was considered, all symptom combinations had 97–100% sensitivity. When exposure history was not considered, the RIDOH criteria and the combination generated by backward elimination had the highest sensitivity: 95% in 0–4 year-olds, 87% in 5–11 year-olds, and 92% in 12–17 year-olds. All combinations had <30% specificity.

**Table 3:** Diagnostic value of symptom screening in children 0–4 years of age.

	No. (%) participants with symptom		p-value	Sensitivity (95% CI)	Specificity (95% CI)	AUC (95% CI)
	Uninfected (n = 115)	Infected (n = 40)				
Known COVID-19 exposure	31 (27.2)	31 (77.5)	<0.001	77.5 (64.6–90.4)	72.8 (64.6–81.0)	0.75 (0.65–0.86)
*Individual symptoms*						
Cough	31 (27.2)	26 (65.0)	<0.001	65.0 (50.2–79.8)	72.8 (64.6–81.0)	0.69 (0.57–0.80)
Fever	61 (53.5)	28 (70.0)	0.069	70.0 (55.8–84.2)	46.5 (37.3–55.6)	0.58 (0.47–0.70)
Congestion/rhinorrhea	25 (21.9)	15 (37.5)	0.053	37.5 (22.5–52.5)	78.1 (70.5–85.7)	0.58 (0.47–0.69)
Dyspnea	13 (11.4)	7 (17.5)	0.32	17.5 (5.7–29.3)	88.6 (82.8–94.4)	0.53 (0.44–0.62)
Diarrhea	19 (16.7)	8 (20.0)	0.63	20.0 (7.6–32.4)	83.3 (76.5–90.2)	0.52 (0.42–0.61)
Fatigue	7 (6.1)	3 (7.5)	0.76	7.5 (0.0–15.7)	93.9 (89.5–98.3)	0.51 (0.44–0.57)
Vomiting^a^	15 (13.2)	3 (7.7)	0.36	7.7 (0.0–16.1)	86.8 (80.6–93.0)	0.47 (0.40–0.55)
*Modified RIDOH probable case definition* ^b^						
Modified RIDOH^c^	88 (77.2)	38 (95.0)	0.012	95.0 (88.2–100.0)	22.8 (15.1–30.5)	0.59 (0.52–0.66)
Modified RIDOH^c^ or known COVID-19 exposure	99 (86.8)	40 (100.0)	0.016	100.0 (100.0–100.0)	13.2 (7.0–19.4)	0.57 (0.54–0.60)
*Backward elimination* ^b^ ^,^ ^d^						
Cough, dyspnea, fever, congestion/rhinorrhea, or diarrhea	87 (76.3)	38 (95.0)	0.009	95.0 (88.2–100.0)	23.7 (15.9–31.5)	0.59 (0.52–0.67)
Cough, dyspnea, fever, congestion/rhinorrhea, diarrhea, or known COVID-19 exposure	98 (86.0)	40 (100.0)	0.012	100.0 (100.0–100.0)	14.0 (7.7–20.4)	0.57 (0.54–0.60)
*CART* ^b^ ^,^ ^d^						
Cough	31 (27.2)	26 (65.0)	<0.001	65.0 (50.2–79.8)	72.8 (64.6–81.0)	0.69 (0.57–0.80)
Cough, fever, or known COVID-19 exposure	93 (81.6)	39 (97.5)	0.013	97.5 (92.7–100.0)	18.4 (11.3–25.5)	0.58 (0.52–0.64)

^a^There is a missing value for one participant.

^b^The participant was considered to be positive for the symptom combination if at least one of the features listed below was present.

^c^Cough (new) or shortness of breath alone, or two of any of the following: fever, fatigue, congestion/rhinorrhea (new), nausea/vomiting, and diarrhea.

^d^Symptom combinations presented here are the ones that maximized specificity without sacrificing sensitivity (for backward elimination) and that maximized AUC (for CART). Combinations differed when COVID-19 exposure was considered vs. when exposure was not considered.

Abbreviations: AUC, area under the receiver operating curve; CART, classification and regression tree; CI, confidence interval; RIDOH, Rhode Island Department of Health.

**Table 4:** Diagnostic value of symptom screening in children 5–11 years of age.

Symptom	No. (%) participants with symptom		p-value	Sensitivity (95% CI)	Specificity (95% CI)	AUC (95% CI)
	Uninfected (n = 99)	Infected (n = 69)				
Known COVID-19 exposure	50 (50.5)	61 (88.4)	<0.001	88.4 (80.9–96.0)	49.5 (39.6–59.3)	0.69 (0.60–0.78)
*Individual symptoms*						
Headache	26 (26.3)	34 (49.3)	0.002	49.3 (37.5–61.1)	73.7 (65.1–82.4)	0.62 (0.51–0.72)
Cough	32 (32.3)	34 (49.3)	0.027	49.3 (37.5–61.1)	67.7 (58.5–76.9)	0.59 (0.48–0.69)
Fever	34 (34.3)	34 (49.3)	0.052	49.3 (37.5–61.1)	65.7 (56.3–75.0)	0.58 (0.47–0.68)
Myalgia	10 (10.1)	18 (26.1)	0.006	26.1 (15.7–36.4)	89.9 (84.0–95.8)	0.58 (0.50–0.66)
Sore throat	23 (23.2)	26 (37.7)	0.043	37.7 (26.2–49.1)	76.8 (68.4–85.1)	0.57 (0.47–0.67)
Fatigue	9 (9.1)	11 (15.9)	0.18	15.9 (7.3–24.6)	90.9 (85.2–96.6)	0.53 (0.46–0.61)
Anosmia or ageusia^a^	2 (2.0)	5 (7.2)	0.098	7.2 (1.1–13.4)	98.0 (95.2–100.0)	0.53 (0.48–0.57)
Congestion/rhinorrhea	26 (26.3)	19 (27.5)	0.85	27.5 (17.0–38.1)	73.7 (65.1–82.4)	0.51 (0.41–0.60)
Dyspnea	5 (5.1)	3 (4.3)	0.83	4.3 (0.0–9.2)	94.9 (90.6–99.3)	0.50 (0.45–0.54)
Diarrhea	9 (9.1)	5 (7.2)	0.67	7.2 (1.1–13.4)	90.9 (85.2–96.6)	0.49 (0.43–0.55)
Abdominal pain	15 (15.2)	6 (8.7)	0.21	8.7 (2.0–15.3)	84.8 (77.8–91.9)	0.47 (0.40–0.54)
Nausea/vomiting	9 (9.1)	0 (0.0)	0.010	0.0 (0.0–0.0)	90.9 (85.2–96.6)	0.46 (0.43–0.48)
*RIDOH probable case definition* ^b^						
RIDOH criteria	81 (81.8)	60 (87.0)	0.37	87.0 (79.0–94.9)	18.2 (10.6–25.8)	0.53 (0.45–0.60)
RIDOH criteria or known COVID-19 exposure	79 (79.8)	67 (97.1)	0.001	97.1 (93.1–100.0)	20.2 (12.3–28.1)	0.53 (0.49–0.57)
*Backward elimination* ^b^ ^,^ ^c^						
Cough, fever, headache, sore throat, myalgia, congestion/rhinorrhea, fatigue, or anosmia or ageusia	73 (73.7)	60 (87.0)	0.053	87.0 (79.0–94.9)	26.3 (17.6–34.9)	0.57 (0.48–0.65)
Known COVID-19 exposure, cough, fever, headache, sore throat, or myalgia	81 (81.8)	68 (98.6)	<0.001	98.6 (95.7–100.0)	18.2 (10.6–25.8)	0.58 (0.53–0.63)
*CART* ^b^ ^,^ ^c^						
Headache, sore throat, or cough	57 (57.6)	54 (78.3)	0.005	78.3 (68.5–88.0)	42.4 (32.7–52.2)	0.60 (0.51–0.70)
Myalgia, anosmia or ageusia, headache, sore throat, congestion/rhinorrhea, or known COVID-19 exposure^a^	77 (77.8)	68 (98.6)	<0.001	98.6 (95.7–100.0)	22.2 (14.0–30.4)	0.60 (0.55–0.66)

^a^There is a missing value for one participant.

^b^The participant was considered to be positive for the symptom combination if at least one of the features listed below was present.

^c^Symptom combinations presented here are the ones that maximized specificity without sacrificing sensitivity (for backward elimination) and that maximized AUC (for CART). Combinations differed when COVID-19 exposure was considered vs. when exposure was not considered.

Abbreviations: AUC, area under the receiver operating curve; CART, classification and regression tree; CI, confidence interval; RIDOH, Rhode Island Department of Health.

**Table 5:** Diagnostic value of symptom screening in adolescents 12–17 years of age.

Symptom	No. (%) participants with symptom		p-value	Sensitivity (95% CI)	Specificity (95% CI)	AUC (95% CI)
	Uninfected (n = 125)	Infected (n = 108)				
Known COVID-19 exposure	69 (55.2)	97 (89.8)	<0.001	89.8 (84.1–95.5)	44.8 (36.1–53.5)	0.67 (0.60–0.75)
*Individual symptoms*						
Cough	40 (32.0)	59 (54.6)	<0.001	55.1 (45.7–64.6)	68.0 (59.8–76.2)	0.61 (0.53–0.70)
Headache	52 (41.6)	63 (58.3)	0.011	58.3 (49.0–67.6)	58.4 (49.8–67.0)	0.58 (0.49–0.67)
Sore throat	36 (28.8)	48 (44.4)	0.013	44.9 (35.4–54.3)	71.2 (63.3–79.1)	0.58 (0.49–0.67)
Congestion/rhinorrhea	25 (20.0)	38 (35.2)	0.009	35.5 (26.4–44.6)	80.0 (73.0–87.0)	0.58 (0.50–0.66)
Myalgia	32 (25.6)	40 (37.0)	0.060	37.0 (27.9–46.1)	74.4 (66.7–82.1)	0.56 (0.47–0.64)
Anosmia or ageusia^a^	7 (5.6)	19 (17.8)	0.004	17.8 (10.5–25.0)	94.4 (90.3–98.4)	0.56 (0.50–0.62)
Fever	35 (28.0)	41 (38.0)	0.11	38.3 (29.1–47.5)	72.0 (64.1–79.9)	0.55 (0.47–0.64)
Fatigue	27 (21.6)	29 (26.9)	0.35	27.1 (18.7–35.5)	78.4 (71.2–85.6)	0.53 (0.45–0.60)
Diarrhea	12 (9.6)	15 (13.9)	0.31	14.0 (7.4–20.6)	90.4 (85.2–95.6)	0.52 (0.46–0.58)
Dyspnea	14 (11.2)	11 (10.2)	0.80	10.3 (4.5–16.0)	88.8 (83.3–94.3)	0.50 (0.44–0.55)
Abdominal pain	20 (16.0)	7 (6.5)	0.024	6.5 (1.9–11.2)	84.0 (77.6–90.4)	0.45 (0.40–0.51)
Nausea/vomiting	22 (17.6)	5 (4.6)	0.002	4.7 (0.7–8.8)	82.4 (75.7–89.1)	0.44 (0.38–0.49)
*RIDOH probable case definition* ^b^						
RIDOH criteria	98 (78.4)	99 (91.7)	0.005	91.7 (86.5–96.9)	21.6 (14.4–28.8)	0.57 (0.50–0.63)
RIDOH criteria or known COVID-19 exposure	104 (83.2)	107 (99.1)	<0.001	99.1 (97.3–100.0)	16.8 (10.2–23.4)	0.54 (0.52–0.57)
*Backward elimination* ^b^ ^,^ ^c^						
Cough, fever, headache, sore throat, myalgia, congestion/rhinorrhea, or anosmia or ageusia	90 (72.0)	99 (91.7)	<0.001	91.7 (86.5–96.9)	28.0 (20.1–35.9)	0.60 (0.53–0.66)
Known COVID-19 exposure, cough, or headache	97 (77.6)	108 (100.0)	<0.001	100.0 (100.0–100.0)	22.4 (15.1–29.7)	0.61 (0.58–0.65)
*CART* ^*b*,*c*^						
Cough, nausea/vomiting, or headache	76 (60.8)	88 (81.5)	<0.001	81.5 (74.2–88.8)	39.2 (30.6–47.8)	0.60 (0.52–0.68)
Cough, fever, sore throat, congestion/rhinorrhea, nausea/vomiting, or known COVID-19 exposure	113 (90.4)	107 (99.1)	0.004	97.1 (93.1–100.0)	14.1 (7.3–21.0)	0.54 (0.51–0.58)

^a^There is a missing value for two participants.

^b^The participant was considered to be positive for the symptom combination if at least one of the features listed below was present.

^c^Symptom combinations presented here are the ones that maximized specificity without sacrificing sensitivity (for backward elimination) and that maximized AUC (for CART). Combinations differed when COVID-19 exposure was considered vs. when exposure was not considered.

Abbreviations: AUC, area under the receiver operating curve; CART, classification and regression tree; CI, confidence interval; RIDOH, Rhode Island Department of Health.

In children <5 years old, fever and cough were the individual symptoms with the highest sensitivity at 70% and 65%, respectively (Table 3). In children 5–11 years old, no individual symptom had >50% sensitivity for COVID-19. Anosmia or ageusia had 98% specificity; dyspnea had 95% specificity (Table 4). Among adolescents 12–17 years old, cough and headache were the individual symptoms with the highest sensitivity. Anosmia or ageusia had 94% specificity (Table 5).

In the sensitivity analysis of participants evaluated with the standardized template before PCR testing, the AUCs of exposure history and individual symptoms were similar to those calculated for the entire study population, with differences of ≤0.02 (S10–S12 Tables).

## Discussion

In this study, we assessed the diagnostic properties of symptoms for SARS-CoV-2 infection in a large pediatric cohort, >90% of whom presented to primary care and were evaluated with a standardized symptom template before PCR testing. We identified symptom combinations with high sensitivity, particularly in conjunction with COVID-19 exposure; however, all symptom combinations had poor specificities. We failed to identify any individual symptom or symptom combination with AUC >0.70, underscoring the importance of widely available SARS-CoV-2 testing with rapid turnaround.

The AUCs observed in our study likely are higher than in the general population for several reasons. First, few study participants were asymptomatic, thus maximizing sensitivity of symptoms. Second, our study took place in the spring and summer; specificities of symptoms are expected to decrease further in the winter as more respiratory viruses circulate. Third, the reliability of COVID-19 exposure history probably was higher in our cohort since many participants were tested during a stay-at-home order. Therefore, they likely had few contacts and were better able to stay informed of the infection status of their contacts.

Reports of pediatric COVID-19 symptoms mostly include hospitalized participants [3, 15–18]. One exception is a study conducted in Alberta, Canada, which used provincial databases to assess the association of symptoms with SARS-CoV-2 PCR positivity [8]. This study found a high positive predictive value for anosmia or ageusia; similarly, we observed a high specificity of these symptoms. Our study differs in a few ways. First, in Alberta, the symptom questionnaire was applied after test results were known, whereas symptoms were assessed before testing in >90% of our cohort, reducing recall bias. Second, we age-stratified participants and detected differences in COVID-19 presentation between age groups. These differences were similar to those reported by the BRAVE study, which evaluated children with a close SARS-CoV-2-infected contact [19]: elementary school-aged children were most likely to have asymptomatic COVID-19 (though the difference did not reach statistical significance in our cohort), the youngest children were most likely to be febrile, and adolescents were more likely to report anosmia or ageusia compared to elementary school-aged children.

Our findings have clinical and public health implications. The low AUCs we observed strongly argue against the use of symptoms to diagnose pediatric COVID-19. However, anosmia or ageusia in children ≥5 years old and dyspnea in children 5–11 years old are highly specific, and their presence should alert providers to quickly isolate and test the patient. Exposure history most accurately predicted SARS-CoV-2 infection and should remain a cornerstone of quarantine recommendations. Because of differences between our cohort and daycare and school attendees, the diagnostic characteristics we observed may not be generalizable to that group. However, our findings suggest that different age groups need distinct symptom screening criteria. Additionally, because of the low specificities of most symptoms, easily accessible tests with rapid turnaround times are critical to minimize unnecessary absences.

With respect to the secondary aim of our study, we identified Hispanic ethnicity as an independent risk factor for COVID-19 compared to the reference group of NH Black. Both the BRAVE study and another study conducted in Washington, DC similarly found significantly higher SARS-CoV-2 positivity in Hispanic children [19, 20]. Further investigation is needed to clarify the contribution of various factors—including multigenerational or multi-family housing, the inability to work from home, and language barriers—to these higher positivity rates [21–25]. The DC study reported that NH Blacks also had higher positivity rates than NH Whites, but we did not detect a difference between these groups, potentially due to insufficient statistical power.

This study had limitations. Data were collected early in the pandemic; however, symptoms are not expected to change over the course of the pandemic, and the clinical and public health implications of this study remain relevant and practical. As previously discussed, the AUCs of exposure and symptoms that we observed may represent a “best case scenario,” but this possibility only strengthens the overarching message that symptoms are poorly predictive of COVID-19. Symptoms were self-reported and may not have been accurate. However, the use of a standardized checklist to query patients and/or their caregivers, rather than asking them to recall symptoms spontaneously, and the elimination of certain symptoms (myalgia, headache, sore throat, abdominal pain, nausea, anosmia, and ageusia) for the youngest age group may have helped minimize inaccuracy. Time from symptom onset to PCR testing was missing for a significant proportion of patients.

## Conclusion

In all ages, exposure history most accurately predicted infection. No symptom or symptom combination had AUC ≥0.70. Anosmia or ageusia is highly specific in children ≥5 years old and dyspnea in children 5–11 years old and should raise providers’ index of suspicion for COVID-19; however, the sensitivity of this symptom is low. Our overall findings underscore the limited diagnostic value of symptoms and the critical need for widely available, efficient testing.

## Supporting information

S1 Table Differences between patients included and excluded in the study. (DOCX)

S2 Table Participants presenting with only one symptom. (DOCX)

S3 Table Age-stratified clinical presentation among 217 SARS-CoV-2-positive participants. (DOCX)

S4 Table Backward elimination, children 0–4 years of age, symptoms only. (DOCX)

S5 Table Backward elimination, children 0–4 years of age, symptoms and exposure. (DOCX)

S6 Table Backward elimination, children 6–11 years of age, symptoms only. (DOCX)

S7 Table Backward elimination, children 6–11 years of age, symptoms and exposure. (DOCX)

S8 Table Backward elimination, children 12–17 years of age, symptoms only. (DOCX)

S9 Table Backward elimination, children 12–17 years of age, symptoms and exposure. (DOCX)

S10 Table Sensitivity analysis of diagnostic value of exposure and individual symptoms in children 0–4 years of age (n = 138). (DOCX)

S11 Table Sensitivity analysis of diagnostic value of exposure and individual symptoms in children 5–11 years of age. (DOCX)

S12 Table Sensitivity analysis of diagnostic value of exposure and individual symptoms in children 12–17 years of age. (DOCX)

S1 Dataset Deidentified original dataset. (XLSX)

## Abbreviations

SARS-CoV-2: severe acute respiratory syndrome coronavirus 2
COVID-19: coronavirus disease 2019
RT-PCR: reverse transcriptase polymerase chain reaction
NH: non-Hispanic
BMI: body mass index
CART: combination generated by classification and regression tree
IQR: interquartile range
AUC: area under the receiver operating curve
