# Supplementary material for: Diagnostic value of symptoms for pediatric SARS-CoV-2 infection in a primary care setting
Source: PLoS One. 2021 Dec 13;16(12):e0249980. doi: 10.1371/journal.pone.0249980 (PMC8668089; doi:10.1371/journal.pone.0249980)
Supplement: S1 Table — (DOCX) [file pone.0249980.s001.docx]

S1 Table: Differences between Patients Included and Excluded in the Study

|  | Excluded (n=248) | Included (n=555) | Total (n=803) | p-value |
| --- | --- | --- | --- | --- |
| Age category |  |  |  | 0.78 |
| 0-4 years | 71 (28.6%) | 154 (27.7%) | 225 (28.0%) |  |
| 5-11 years | 69 (27.8%) | 168 (30.3%) | 237 (29.5%) |  |
| 12-17 years | 108 (43.5%) | 233 (42.0%) | 341 (42.5%) |  |
| Race/ethnicity |  |  |  | <0.001 |
| Hispanic | 153 (61.7%) | 459 (82.7%) | 612 (76.2%) |  |
| NH Black | 28 (11.3%) | 26 (4.7%) | 54 (6.7%) |  |
| NH White | 15 (6.0%) | 30 (5.4%) | 45 (5.6%) |  |
| NH Other | 52 (21.0%) | 40 (7.2%) | 92 (11.5%) |  |
| Uninsured | 32 (12.9%) | 37 (6.7%) | 69 (8.6%) | 0.004 |
| SARS-CoV-2 PCR-positive | 38 (15.3%) | 217 (39.1%) | 255 (31.8%) | <0.001 |

Of 52 excluded patients in the NH Other category, 5 (9.6%) were Asian; 6 (11.5%), >1 race; 1 (1.9%), Pacific Islander; 40 (76.9%), Unknown. Of 40 included patients in the NH Other category, 9 (22.5%) were Asian; 8 (20.0%), >1 race; 1 (2.5%), Pacific Islander; 22 (55.0%), Unknown.
