# Supplementary material for: Diagnostic value of symptoms for pediatric SARS-CoV-2 infection in a primary care setting
Source: PLoS One. 2021 Dec 13;16(12):e0249980. doi: 10.1371/journal.pone.0249980 (PMC8668089; doi:10.1371/journal.pone.0249980)
Supplement: S2 Table — (DOCX) [file pone.0249980.s002.docx]

S2 Table: Participants Presenting with Only One Symptom

|  | No. (%) participants | |
| --- | --- | --- |
|  | Uninfected (n=72) | Infected (n=28) |
| Symptom | | |
| Fever | 22 (30.6) | 11 (33.3) |
| Fatigue | 2 (2.8) | 0 (0.0) |
| Myalgia | 5 (6.9) | 2 (7.1) |
| Headache | 5 (6.9) | 3 (10.7) |
| Cough | 11 (15.3) | 6 (21.4) |
| Dyspnea | 3 (4.2) | 1 (3.6) |
| Sore throat | 3 (4.2) | 3 (10.7) |
| Congestion/rhinorrhea | 9 (12.5) | 1 (3.6) |
| Anosmia or ageusia | 2 (2.8) | 0 (0.0) |
| Abdominal pain | 1 (1.4) | 0 (0.0) |
| Nausea/vomiting | 4 (5.6) | 0 (0.0) |
| Diarrhea | 5 (6.9) | 1 (3.6) |
| Known COVID-19 exposure | 33 (45.8) | 24 (85.7) |
