# Supplementary material for: Diagnostic value of symptoms for pediatric SARS-CoV-2 infection in a primary care setting
Source: PLoS One. 2021 Dec 13;16(12):e0249980. doi: 10.1371/journal.pone.0249980 (PMC8668089; doi:10.1371/journal.pone.0249980)
Supplement: S3 Table — (DOCX) [file pone.0249980.s003.docx]

S3 Table: Age-Stratified Clinical Presentation among 217 SARS-CoV-2-Positive Participants

|  | Total (n=217) | | 0-4 years (n=40) | | 5-11 years (n=69) | | 12-17 years (n=108) | | *P*-value |
| --- | --- | --- | --- | --- | --- | --- | --- | --- | --- |
|  | No. | % (95% CI) | No. | % (95% CI) | No. | % (95% CI) | No. | % (95% CI) |  |
| No symptoms | 20 | 9.2 (5.4, 13.1) | 2 | 5.0 (0.0, 11.8) | 9 | 13.0 (5.1, 21.0) | 9 | 8.3 (3.1, 13.5) | 0.34 |
|  |  |  |  |  |  |  |  |  |  |
| Fever | 103 | 47.5 (40.8, 54.1) | 28 | 70.0 (55.8, 84.2) | 34 | 49.3 (37.5, 61.1) | 41 | 38.0 (28.8, 47.1) | 0.002 |
| Fatigue | 43 | 19.8 (14.5, 25.1) | 3 | 7.5 (0.0, 15.7) | 11 | 15.9 (7.3, 24.6) | 29 | 26.9 (18.5, 35.2) | 0.020 |
| Myalgia | NA |  | NA |  | 18 | 26.1 (15.7, 36.4) | 40 | 37.0 (27.9, 46.1) | 0.13 |
| Headache | NA |  | NA |  | 34 | 49.3 (37.5, 61.1) | 63 | 58.3 (49.0, 67.6) | 0.24 |
| Cough | 119 | 54.8 (48.2, 61.5) | 26 | 65.0 (50.2, 79.8) | 34 | 49.3 (37.5, 61.1) | 59 | 54.6 (45.2, 64.0) | 0.28 |
| Dyspnea | 21 | 9.7 (5.7, 13.6) | 7 | 17.5 (5.7, 29.3) | 3 | 4.3 (0.0, 9.2) | 11 | 10.2 (4.5, 15.9) | 0.079 |
| Sore throat | NA |  | NA |  | 26 | 37.7 (26.2, 49.1) | 48 | 44.4 (35.1, 53.8) | 0.37 |
| Congestion/rhinorrhea | 72 | 33.2 (26.9, 39.4) | 15 | 37.5 (22.5, 52.5) | 19 | 27.5 (17.0, 38.1) | 38 | 35.2 (26.2, 44.2) | 0.47 |
| Anosmia/ageusia | NA |  | NA |  | 5 | 7.2 (1.1, 13.4) | 19 | 17.6 (10.4, 24.8) | 0.047 |
| Abdominal pain | NA |  | NA |  | 6 | 8.7 (2.0, 15.3) | 7 | 6.5 (1.8, 11.1) | 0.58 |
| Nausea/vomiting | 8 | 3.7 (1.2, 6.2) | 3 | 7.6 (0.0, 15.7) | 0 | 0.0 (0.0, 0.0) | 5 | 4.6 (0.7, 8.6) | 0.098 |
| Diarrhea | 28 | 12.9 (8.4, 17.4) | 8 | 20.0 (7.6, 32.4) | 5 | 7.2 (1.1, 13.4) | 15 | 13.9 (7.4, 20.4) | 0.15 |

Myalgia, headache, sore throat, abdominal pain, nausea, and anosmia/ageusia were not assessed in children 0-4 years-old due to the lower reliability of these symptoms in this group.

Abbreviations: CI, confidence interval; HEENT, head/eyes/ears/nose/throat; NA, not applicable.
