# Supplementary material for: Diagnostic value of symptoms for pediatric SARS-CoV-2 infection in a primary care setting
Source: PLoS One. 2021 Dec 13;16(12):e0249980. doi: 10.1371/journal.pone.0249980 (PMC8668089; doi:10.1371/journal.pone.0249980)
Supplement: S4 Table — (DOCX) [file pone.0249980.s004.docx]

S4 Table: Backward Elimination, Children 0-4 Years of Age, Symptoms Only

| Symptom(s) removed | No. (%) participants with symptom | | p-value | Sensitivity  (95% CI) | Specificity  (95% CI) | AUC |
| --- | --- | --- | --- | --- | --- | --- |
|  | Uninfected (n=115) | Infected (n=40) |  |  |  |  |
| None | 88 (77.2) | 38 (95.0) | 0.012 | 95.0 (88.2-100.0) | 22.8 (15.1-30.5) | 0.59 |
| Vomiting | 87 (76.3) | 38 (95.0) | 0.009 | 95.0 (88.2-100.0) | 23.7 (15.9-31.5) | 0.59 |
| Vomiting + fatigue | 87 (76.3) | 38 (95.0) | 0.009 | 95.0 (88.2-100.0) | 23.7 (15.9-31.5) | 0.59 |
| Vomiting + fatigue + diarrhea | 83 (72.8) | 37 (92.5) | 0.010 | 92.5 (84.3-100.0) | 27.2 (19.0-35.4) | 0.60 |

Abbreviations: AUC, area under the receiver operating curve; CI, confidence interval.
