# Supplementary material for: Diagnostic value of symptoms for pediatric SARS-CoV-2 infection in a primary care setting
Source: PLoS One. 2021 Dec 13;16(12):e0249980. doi: 10.1371/journal.pone.0249980 (PMC8668089; doi:10.1371/journal.pone.0249980)
Supplement: S5 Table — (DOCX) [file pone.0249980.s005.docx]

S5 Table: Backward Elimination, Children 0-4 Years of Age, Symptoms and Exposure

| Symptom(s) removed | No. (%) participants with symptom | | p-value | Sensitivity  (95% CI) | Specificity  (95% CI) | AUC |
| --- | --- | --- | --- | --- | --- | --- |
|  | Uninfected (n=115) | Infected (n=40) |  |  |  |  |
| None | 99 (86.8) | 40 (100.0) | 0.016 | 100.0 (100.0-100.0) | 13.2 (7.0-19.4) | 0.57 |
| Vomiting | 98 (86.0) | 40 (100.0) | 0.012 | 100.0 (100.0-100.0) | 14.0 (7.7-20.4) | 0.57 |
| Vomiting + fatigue | 98 (86.0) | 40 (100.0) | 0.012 | 100.0 (100.0-100.0) | 14.0 (7.7-20.4) | 0.57 |
| Vomiting + fatigue + diarrhea | 96 (84.2) | 39 (97.5) | 0.028 | 97.5 (92.7-100.0) | 15.8 (9.1-22.5) | 0.57 |

Abbreviations: AUC, area under the receiver operating curve; CI, confidence interval.
