# Supplementary material for: Diagnostic value of symptoms for pediatric SARS-CoV-2 infection in a primary care setting
Source: PLoS One. 2021 Dec 13;16(12):e0249980. doi: 10.1371/journal.pone.0249980 (PMC8668089; doi:10.1371/journal.pone.0249980)
Supplement: S6 Table — (DOCX) [file pone.0249980.s006.docx]

S6 Table: Backward Elimination, Children 6-11 Years of Age, Symptoms Only

| Symptom(s) removed | No. (%) participants with symptom | | p-value | Sensitivity  (95% CI) | Specificity  (95% CI) | AUC |
| --- | --- | --- | --- | --- | --- | --- |
|  | Uninfected (n=99) | Infected (n=69) |  |  |  |  |
| None | 81 (81.8) | 60 (87.0) | 0.37 | 87.0 (79.0-94.9) | 18.2 (10.6-25.8) | 0.53 |
| Nausea/vomiting | 78 (78.8) | 60 (87.0) | 0.17 | 87.0 (79.0-94.9) | 21.2 (13.2-29.3) | 0.54 |
| Nausea/vomiting + abdominal pain | 77 (77.8) | 60 (87.0) | 0.13 | 87.0 (79.0-94.9) | 22.2 (14.0-30.4) | 0.55 |
| Nausea/vomiting + abdominal pain + diarrhea | 74 (74.7) | 60 (87.0) | 0.053 | 87.0 (79.0-94.9) | 25.3 (16.7-33.8) | 0.56 |
| Nausea/vomiting + abdominal pain + diarrhea + dyspnea | 73 (73.7) | 60 (87.0) | 0.053 | 87.0 (79.0-94.9) | 26.3 (17.6-34.9) | 0.57 |
| Nausea/vomiting + abdominal pain + diarrhea + dyspnea + congestion/rhinorrhea | 68 (68.7) | 59 (85.5) | 0.013 | 85.5 (77.2-93.8) | 31.3 (22.2-40.4) | 0.58 |

Abbreviations: AUC, area under the receiver operating curve; CI, confidence interval.
