# Supplementary material for: Diagnostic value of symptoms for pediatric SARS-CoV-2 infection in a primary care setting
Source: PLoS One. 2021 Dec 13;16(12):e0249980. doi: 10.1371/journal.pone.0249980 (PMC8668089; doi:10.1371/journal.pone.0249980)
Supplement: S7 Table — (DOCX) [file pone.0249980.s007.docx]

S7 Table: Backward Elimination, Children 6-11 Years of Age, Symptoms and Exposure

| Symptom(s) removed | No. (%) participants with symptom | | p-value | Sensitivity  (95% CI) | Specificity  (95% CI) | AUC |
| --- | --- | --- | --- | --- | --- | --- |
|  | Uninfected (n=99) | Infected (n=69) |  |  |  |  |
| None | 92 (92.9) | 68 (98.6) | 0.092 | 98.6 (95.7-100.0) | 7.1 (2.0-12.1) | 0.53 |
| Nausea/vomiting | 89 (89.9) | 68 (98.6) | 0.026 | 98.6 (95.7-100.0) | 10.1 (4.2-16.0) | 0.54 |
| Nausea/vomiting + abdominal pain | 88 (88.9) | 68 (98.6) | 0.017 | 98.6 (95.7-100.0) | 11.1 (4.9-17.3) | 0.55 |
| Nausea/vomiting + abdominal pain + diarrhea | 85 (85.9) | 68 (98.6) | 0.005 | 98.6 (95.7-100.0) | 14.1 (7.3-21.0) | 0.56 |
| Nausea/vomiting + abdominal pain + diarrhea + dyspnea | 84 (84.8) | 68 (98.6) | 0.005 | 98.6 (95.7-100.0) | 15.2 (8.1-22.2) | 0.57 |
| Nausea/vomiting + abdominal pain + diarrhea + shortness of breath + congestion/rhinorrhea | 82 (82.8) | 68 (98.6) | 0.001 | 98.6 (95.7-100.0) | 17.2 (9.7-24.6) | 0.58 |
| Nausea/vomiting + abdominal pain + diarrhea + dyspnea + congestion/rhinorrhea + anosmia/ageusia | 82 (82.8) | 68 (98.6) | <0.001 | 98.6 (95.7-100.0) | 17.2 (9.7-24.6) | 0.58 |
| Nausea/vomiting + abdominal pain + diarrhea + dyspnea + congestion/rhinorrhea + anosmia/ageusia + fatigue | 81 (81.8) | 68 (98.6) | <0.001 | 98.6 (95.7-100.0) | 18.2 (10.6-25.8) | 0.58 |
| Nausea/vomiting + abdominal pain + diarrhea + dyspnea + congestion/rhinorrhea + anosmia/ageusia + fatigue + sore throat | 79 (79.8) | 67 (97.1) | 0.001 | 97.1 (93.1-100.0) | 20.2 (12.3-28.1) | 0.59 |

Abbreviations: AUC, area under the receiver operating curve; CI, confidence interval.
