# Supplementary material for: Diagnostic value of symptoms for pediatric SARS-CoV-2 infection in a primary care setting
Source: PLoS One. 2021 Dec 13;16(12):e0249980. doi: 10.1371/journal.pone.0249980 (PMC8668089; doi:10.1371/journal.pone.0249980)
Supplement: S8 Table — (DOCX) [file pone.0249980.s008.docx]

S8 Table: Backward Elimination, Children 12-17 Years of Age, Symptoms Only

| Symptom(s) removed | No. (%) participants with symptom | | p-value | Sensitivity  (95% CI) | Specificity  (95% CI) | AUC |
| --- | --- | --- | --- | --- | --- | --- |
|  | Uninfected (n=125) | Infected (n=108) |  |  |  |  |
| None | 99 (79.2) | 99 (91.7) | 0.008 | 91.7 (86.5-96.9) | 20.8 (13.7-27.9) | 0.56 |
| Nausea/vomiting | 99 (79.2) | 99 (91.7) | 0.008 | 91.7 (86.5-96.9) | 20.8 (13.7-27.9) | 0.56 |
| Nausea/vomiting + abdominal pain | 94 (75.2) | 99 (91.7) | <0.001 | 91.7 (86.5-96.9) | 24.8 (17.2-32.4) | 0.58 |
| Nausea/vomiting + abdominal pain + dyspnea | 94 (75.2) | 99 (91.7) | <0.001 | 91.7 (86.5-96.9) | 24.8 (17.2-32.4) | 0.58 |
| Nausea/vomiting + abdominal pain + dyspnea + diarrhea | 92 (73.6) | 99 (91.7) | <0.001 | 91.7 (86.5-96.9) | 26.4 (18.7-34.1) | 0.59 |
| Nausea/vomiting + abdominal pain + dyspnea + diarrhea + fatigue | 90 (72.0) | 99 (91.7) | <0.001 | 91.7 (86.5-96.9) | 28.0 (20.1-35.9) | 0.60 |
| Nausea/vomiting + abdominal pain + dyspnea + diarrhea + fatigue + fever* | 87 (69.6) | 98 (90.7) | <0.001 | 90.7 (85.3-96.2) | 30.4 (22.3-38.5) | 0.61 |

Abbreviations: AUC, area under the receiver operating curve; CI, confidence interval.
