# Supplementary material for: Diagnostic value of symptoms for pediatric SARS-CoV-2 infection in a primary care setting
Source: PLoS One. 2021 Dec 13;16(12):e0249980. doi: 10.1371/journal.pone.0249980 (PMC8668089; doi:10.1371/journal.pone.0249980)
Supplement: S9 Table — (DOCX) [file pone.0249980.s009.docx]

S9 Table: Backward Elimination, Children 12-17 Years of Age, Symptoms and Exposure

| Symptom(s) removed | No. (%) participants with symptom | | p-value | Sensitivity  (95% CI) | Specificity  (95% CI) | AUC |
| --- | --- | --- | --- | --- | --- | --- |
|  | Uninfected (n=125) | Infected (n=108) |  |  |  |  |
| None | 115 (92.0) | 108 (100.0) | 0.003 | 100.0 (100.0-100.0) | 8.0 (3.2-12.8) | 0.54 |
| Nausea/vomiting | 115 (92.0) | 108 (100.0) | 0.003 | 100.0 (100.0-100.0) | 8.0 (3.2-12.8) | 0.54 |
| Nausea/vomiting + abdominal pain | 110 (88.0) | 108 (100.0) | <0.001 | 100.0 (100.0-100.0) | 12.0 (6.3-17.7) | 0.56 |
| Nausea/vomiting + abdominal pain + dyspnea | 110 (88.0) | 108 (100.0) | <0.001 | 100.0 (100.0-100.0) | 12.0 (6.3-17.7) | 0.56 |
| Nausea/vomiting + abdominal pain + dyspnea + diarrhea | 108 (86.4) | 108 (100.0) | <0.001 | 100.0 (100.0-100.0) | 13.6 (7.6-19.6) | 0.57 |
| Nausea/vomiting + abdominal pain + dyspnea + diarrhea + fatigue | 107 (85.6) | 108 (100.0) | <0.001 | 100.0 (100.0-100.0) | 14.4 (8.2-20.6) | 0.57 |
| Nausea/vomiting + abdominal pain + dyspnea + diarrhea + fatigue + fever^a^ | 106 (84.8) | 108 (100.0) | <0.001 | 100.0 (100.0-100.0) | 15.2 (8.9-21.5) | 0.58 |
| Nausea/vomiting + abdominal pain + dyspnea + diarrhea + fatigue + fever + anosmia/ageusia | 106 (84.8) | 108 (100.0) | <0.001 | 100.0 (100.0-100.0) | 15.2 (8.9-21.5) | 0.58 |
| Nausea/vomiting + abdominal pain + dyspnea + diarrhea + fatigue + fever + anosmia/ageusia + myalgia | 104 (83.2) | 108 (100.0) | <0.001 | 100.0 (100.0-100.0) | 16.8 (10.2-23.4) | 0.58 |
| Nausea/vomiting + abdominal pain + dyspnea + diarrhea + fatigue + fever + anosmia/ageusia + myalgia + congestion/rhinorrhea | 102 (81.6) | 108 (100.0) | <0.001 | 100.0 (100.0-100.0) | 18.4 (11.6-25.2) | 0.59 |
| Nausea/vomiting + abdominal pain + dyspnea + diarrhea + fatigue + fever + anosmia/ageusia + myalgia + congestion/rhinorrhea  + sore throat | 97 (77.6) | 108 (100.0) | <0.001 | 100.0 (100.0-100.0) | 22.4 (15.1-29.7) | 0.61 |
| Nausea/vomiting + abdominal pain + dyspnea + diarrhea + fatigue + fever + anosmia/ageusia + myalgia + congestion/rhinorrhea  + sore throat + headache | 82 (65.6) | 106 (98.1) | <0.001 | 98.1 (95.6-100.0) | 34.4 (26.1-42.7) | 0.66 |

^a^There is a missing value for one participant.

Abbreviations: AUC, area under the receiver operating curve; CI, confidence interval.
