# Supplementary material for: Diagnostic value of symptoms for pediatric SARS-CoV-2 infection in a primary care setting
Source: PLoS One. 2021 Dec 13;16(12):e0249980. doi: 10.1371/journal.pone.0249980 (PMC8668089; doi:10.1371/journal.pone.0249980)
Supplement: S10 Table — (DOCX) [file pone.0249980.s010.docx]

S10 Table: Sensitivity Analysis of Diagnostic Value of Exposure and Individual Symptoms in Children 0-4 Years of Age (n=138)

|  | No. (%) participants with symptom | | p-value | Sensitivity  (95% CI) | Specificity  (95% CI) | AUC |
| --- | --- | --- | --- | --- | --- | --- |
|  | Uninfected (n=96) | Infected (n=37) |  |  |  |  |
| Known COVID-19 exposure | 29 (30.2) | 28 (75.7) | <0.001 | 75.7 (61.9-89.5) | 69.8 (60.6-79.0) | 0.73 |
|  |  |  |  |  |  |  |
| *Individual symptoms* | | | | | | |
| Cough | 29 (30.2) | 25 (67.6) | <0.001 | 67.6 (52.5-82.7) | 69.8 (60.6-79.0) | 0.69 |
| Congestion/rhinorrhea | 22 (22.9) | 15 (40.5) | 0.042 | 40.5 (24.7-56.4) | 77.3 (69.0-85.7) | 0.59 |
| Fever | 58 (60.4) | 27 (73.0) | 0.18 | 73.0 (58.7-87.3) | 39.2 (29.5-48.9) | 0.56 |
| Dyspnea | 11 (11.5) | 5 (13.5) | 0.74 | 13.5 (2.5-24.5) | 88.5 (82.2-94.9) | 0.51 |
| Diarrhea | 19 (19.8) | 8 (21.6) | 0.81 | 21.6 (8.4-34.9) | 80.2 (72.2-88.2) | 0.51 |
| Fatigue | 7 (7.3) | 3 (8.1) | 0.87 | 8.1 (0.0-16.9) | 92.7 (87.5-97.9) | 0.50 |
| Vomiting^a^ | 15 (15.6) | 3 (8.3) | 0.28 | 8.3 (0.0-17.4) | 84.4 (77.1-91.6) | 0.46 |

^a^There is a missing value for one participant.

^b^Cough or shortness of breath alone, or two of any of the following: fever, fatigue, congestion/rhinorrhea, nausea/vomiting, and diarrhea.

Abbreviations: AUC, area under the receiver operating curve; CI, confidence interval.
