# Supplementary material for: Diagnostic value of symptoms for pediatric SARS-CoV-2 infection in a primary care setting
Source: PLoS One. 2021 Dec 13;16(12):e0249980. doi: 10.1371/journal.pone.0249980 (PMC8668089; doi:10.1371/journal.pone.0249980)
Supplement: S11 Table — (DOCX) [file pone.0249980.s011.docx]

S11 Table: Sensitivity Analysis of Diagnostic Value of Exposure and Individual Symptoms in Children 5-11 Years of Age

| Symptom | No. (%) participants with symptom | | p-value | Sensitivity | Specificity | AUC |
| --- | --- | --- | --- | --- | --- | --- |
|  | Uninfected (n=91) | Infected (n=67) |  |  |  |  |
| Known COVID-19 exposure | 50 (54.9) | 60 (89.6) | <0.001 | 89.6 (82.2-96.9) | 45.1 (34.8-55.3) | 0.67 |
|  |  |  |  |  |  |  |
| *Individual symptoms* | | | | | | |
| Headache | 25 (27.5) | 33 (49.3) | 0.005 | 49.3 (37.3-61.2) | 73.1 (64.1-82.1) | 0.61 |
| Cough | 32 (35.2) | 34 (50.7) | 0.050 | 50.7 (38.8-62.7) | 64.8 (55.0-74.6) | 0.58 |
| Myalgia | 10 (11.0) | 18 (26.9) | 0.010 | 26.9 (16.3-37.5) | 89.0 (82.6-95.4) | 0.58 |
| Fever | 33 (36.3) | 33 (49.3) | 0.102 | 49.3 (37.3-61.2) | 63.7 (53.9-73.6) | 0.57 |
| Sore throat | 23 (25.3) | 26 (38.8) | 0.069 | 38.8 (27.1-50.5) | 74.7 (65.8-83.7) | 0.57 |
| Fatigue | 9 (9.7) | 11 (16.4) | 0.20 | 16.4 (7.5-25.3) | 90.1 (84.0-96.2) | 0.53 |
| Anosmia/ageusia^a^ | 2 (2.2) | 5 (7.5) | 0.12 | 7.5 (1.2-13.8) | 97.8 (94.7-100.0) | 0.53 |
| Congestion/rhinorrhea | 26 (28.6) | 19 (28.4) | 0.98 | 28.4 (17.6-39.2) | 71.4 (62.1-80.7) | 0.50 |
| Dyspnea | 4 (4.4) | 3 (4.5) | 0.98 | 4.5 (0.0-9.4) | 95.6 (91.4-99.8) | 0.50 |
| Diarrhea | 9 (9.9) | 5 (7.5) | 0.60 | 7.5 (1.2-13.8) | 90.1 (84.0-96.2) | 0.49 |
| Abdominal pain | 14 (15.4) | 5 (7.5) | 0.13 | 7.5 (1.2-13.8) | 84.6 (77.2-92.0) | 0.46 |
| Nausea/vomiting | 9 (9.9) | 0 (0.0) | 0.008 | 0.0 (0.0-0.0) | 90.1 (84.0-96.2) | 0.45 |

^a^There is a missing value for one participant.

Abbreviations: AUC, area under the receiver operating curve; CI, confidence interval.
