# Supplementary material for: Diagnostic value of symptoms for pediatric SARS-CoV-2 infection in a primary care setting
Source: PLoS One. 2021 Dec 13;16(12):e0249980. doi: 10.1371/journal.pone.0249980 (PMC8668089; doi:10.1371/journal.pone.0249980)
Supplement: S12 Table — (DOCX) [file pone.0249980.s012.docx]

S12 Table: Sensitivity Analysis of Diagnostic Value of Exposure and Individual Symptoms in Children 12-17 Years of Age

| Symptom | No. (%) participants with symptom | | p-value | Sensitivity | Specificity | AUC |
| --- | --- | --- | --- | --- | --- | --- |
|  | Uninfected (n=114) | Infected (n=106) |  |  |  |  |
| Known COVID-19 exposure | 68 (59.6) | 95 (89.6) | <0.001 | 89.6 (83.8-95.4) | 40.4 (31.3-49.4) | 0.65 |
|  |  |  |  |  |  |  |
| *Individual symptoms* | | | | | | |
| Cough | 39 (34.2) | 58 (54.7) | 0.002 | 54.7 (45.2-64.2) | 65.8 (57.1-74.5) | 0.60 |
| Headache | 51 (44.7) | 63 (59.4) | 0.029 | 59.4 (50.1-68.8) | 55.3 (46.1-64.4) | 0.57 |
| Congestion/rhinorrhea | 25 (21.9) | 38 (35.8) | 0.022 | 35.8 (26.7-45.0) | 78.1 (70.5-85.7) | 0.57 |
| Sore throat | 36 (31.6) | 47 (44.3) | 0.051 | 44.3 (34.9-53.8) | 68.4 (59.9-77.0) | 0.56 |
| Myalgia | 32 (28.1) | 40 (37.7) | 0.13 | 37.7 (28.5-47.0) | 71.9 (63.7-80.2) | 0.55 |
| Anosmia/ageusia^a^ | 7 (6.1) | 19 (18.1) | 0.007 | 18.1 (10.7-25.5) | 93.8 (89.4-98.2) | 0.56 |
| Fever | 34 (29.8) | 41 (38.7) | 0.17 | 38.7 (29.4-48.0) | 70.2 (61.8-78.6) | 0.54 |
| Fatigue | 26 (22.8) | 29 (27.4) | 0.44 | 27.4 (18.9-35.8) | 77.2 (69.5-84.9) | 0.52 |
| Diarrhea | 11 (9.6) | 15 (14.2) | 0.30 | 14.2 (7.5-20.8) | 90.4 (84.9-95.8) | 0.52 |
| Dyspnea | 13 (11.4) | 11 (10.4) | 0.81 | 10.4 (4.6-16.2) | 88.6 (82.8-94.4) | 0.50 |
| Abdominal pain | 15 (13.2) | 7 (6.6) | 0.11 | 6.6 (1.9-11.3) | 86.8 (80.6-93.0) | 0.47 |
| Nausea/vomiting | 19 (16.7) | 5 (4.7) | 0.005 | 4.7 (0.1-8.8) | 83.3 (76.5-90.2) | 0.44 |

^a^There is a missing value for two participants.

Abbreviations: AUC, area under the receiver operating curve; CI, confidence interval
